# Beyond the evoked/intrinsic neural process dichotomy

**DOI:** 10.1162/NETN_a_00028

**Published:** 2018-03-01

**Authors:** Taylor Bolt, Michael L. Anderson, Lucina Q. Uddin

**Affiliations:** Department of Psychology, University of Miami, Coral Gables, FL, USA; Department of Philosophy and Brain and Mind Institute, Western University, London, ON, Canada; Institute for Advanced Computer Studies, Program in Neuroscience and Cognitive Science, University of Maryland, College Park, MD, USA; Neuroscience Program, University of Miami Miller School of Medicine, Miami, FL, USA

**Keywords:** Intrinsic activity, Task-evoked activity, Enabling constraint, Synergy, Neural variability

## Abstract

Contemporary functional neuroimaging research has increasingly focused on characterization of intrinsic or “spontaneous” brain activity. Analysis of intrinsic activity is often contrasted with analysis of task-evoked activity that has traditionally been the focus of cognitive neuroscience. But does this evoked/intrinsic dichotomy adequately characterize human brain function? Based on empirical data demonstrating a close functional interdependence between intrinsic and task-evoked activity, we argue that the dichotomy between intrinsic and task-evoked activity as unobserved contributions to brain activity is artificial. We present an alternative picture of brain function in which the brain’s spatiotemporal dynamics do not consist of separable intrinsic and task-evoked components, but reflect the enaction of a system of mutual constraints to move the brain into and out of task-appropriate functional configurations. According to this alternative picture, cognitive neuroscientists are tasked with describing both the temporal trajectory of brain activity patterns *across time*, and the modulation of this trajectory by task states, without separating this process into intrinsic and task-evoked components. We argue that this alternative picture of brain function is best captured in a novel explanatory framework called *enabling constraint*. Overall, these insights call for a reconceptualization of functional brain activity, and should drive future methodological and empirical efforts.

## THE TASK-EVOKED/INTRINSIC DIVIDE IN FUNCTIONAL NEUROIMAGING

Describing the brain’s spontaneous or intrinsic activity has increasingly become a central focus of contemporary functional neuroimaging research. Much progress has been made in recent decades demonstrating that the brain’s spontaneous activity is not the result of “noise” or an epiphenomenon of neuronal circuitry, but is crucially relevant to brain function in visual, auditory, and cognitive processes (Busch & VanRullen, 2010; Kenet, Bibitchkov, Tsodyks, Grinvald, & Arieli, 2003; McCormick, 1999; Penn, Riquelme, Feller, & Shatz, 1998; Röschke & Başar, 1988; Tritsch, Yi, Gale, Glowatzki, & Bergles, 2007). More recently, spontaneous/intrinsic blood oxygen level dependent (BOLD) activity recorded by functional magnetic resonance imaging (fMRI) has been shown to reflect functionally significant neural activity related to cognition and behavior, contrary to the original conception of spontaneous BOLD activity as noise in task-based neuroimaging studies (Biswal, Zerrin Yetkin, Haughton, & Hyde, 1995; Buckner, Krienen, Castellanos, Diaz, & Yeo, 2011; Fox & Raichle, 2007; Fox, Snyder, Zacks, & Raichle, 2006; Fransson, 2005; Smith et al., 2009). The increasing recognition of spontaneous or intrinsic activity as functionally relevant to cognition and behavior introduces the challenge of producing an account of how spontaneous/intrinsic activity and stimulus-driven/evoked activity interact.

This review is organized into four sections. In the first section, we discuss accounts of brain activity in terms of a dichotomy of intrinsic and task-evoked activity. These accounts divide brain activity into independent intrinsic and stimulus-driven components, corresponding to both separate brain functions and separate components of the neural signal that combine linearly to form the observed signal (i.e., the linear superposition principle). Together, these accounts form what we term the task-evoked/intrinsic divide in functional neuroimaging.

In the second section, we challenge the dichotomy between intrinsic and task-evoked activity as two sources of the observed neural signal. We provide empirical support for an alternative account of brain function, arguing that these two forms of activity reflect a single functional process. First, we review recent evidence that intrinsic and task-evoked activity demonstrate similar temporal and spatial properties. Second, we provide an overview of empirical studies that show variability reductions in neuronal signals in response to task stimuli, and challenge the notion that there are separate intrinsic and task-evoked components of the neural signal that linearly sum together to form the recorded signal.

In the third section, we argue that these two streams of neuroscientific research suggest that the distinction between intrinsic and task-evoked activity is artificial. Rather, we suggest a new way forward that pushes one beyond an evoked/intrinsic dichotomy toward a unified picture whereby dynamic brain states enact variability-reducing synergies, such that the brain’s activity space is *constrained* to enable certain functions and suppress others. We suggest that this dynamical process is best captured by methodological techniques that describe and explain this spatiotemporal trajectory of brain activity *across time*, rather than dividing this dynamic into intrinsic and task-evoked components. We then place this dynamical account within a predictive-processing theory of brain function and provide a mechanistic account of our theory in terms of attractor states.

In the fourth section, we argue that this *systems-level temporal* description of brain function is most clearly understood in terms of a novel explanatory framework, known as *enabling constraint* (Anderson, 2014). We give a detailed description of this explanatory framework, and highlight the differences between this and the current, dominant explanatory framework in cognitive neuroscience. While this review is predominantly focused on functional neuroimaging in humans, we highlight areas of convergence with other areas of neuroscientific research in animal models, single-cell and multiunit recordings, and computational modeling, where applicable.

### The Dichotomy of Intrinsic and Task-Evoked Activity

Conventional functional neuroimaging experimental designs are composed of periods of stimulus presentation, interspersed with periods of rest or fixation. Neural activity recorded during stimulus presentations is typically labeled task-evoked or stimulus-driven, while neural activity recorded during rest or nonstimulus periods is typically labeled intrinsic or resting activity. In the past decade, the study of rest or nonstimulus periods has been extended into a research paradigm known as resting-state neuroimaging (resting-state fMRI, resting-state EEG, etc.). The discovery of spatial and temporal structure in the neural activity recorded during nonstimulus blocks (Raichle et al., 2001) and resting-state scans (Biswal et al., 1995; Greicius, Krasnow, Reiss, & Menon, 2003) has produced debate over the functional significance of intrinsic activity and the use of these rest or nonstimulus periods as a baseline for task fMRI studies (Buckner & Vincent, 2007; Gusnard & Raichle, 2001; Morcom & Fletcher, 2007). However, the justification of compartmentalizing the recorded neural signal into intrinsic and task-evoked components is rarely addressed. Neural signals recorded from functional neuroimaging techniques provide no neural marker of the difference between intrinsic and task-evoked neural activity. Rather, this is an experimenter inference about the *latent* sources of the *observed* neural signal in terms of two separate components. Whether this dichotomy of intrinsic and task-evoked activity is respected by the brain needs conceptual and empirical support. Here, we describe the supposed separation of intrinsic and task-evoked activity as supported by the linear superposition principle, and the inference that these represent separate functional processes. Below (see Evidence Challenging the Dichotomy of Intrinsic and Task-Evoked Activity), we describe why we believe this view to be misguided.

### Previous Evidence for the Intrinsic and Task-Evoked Activity Dichotomy

**Figure 1.**
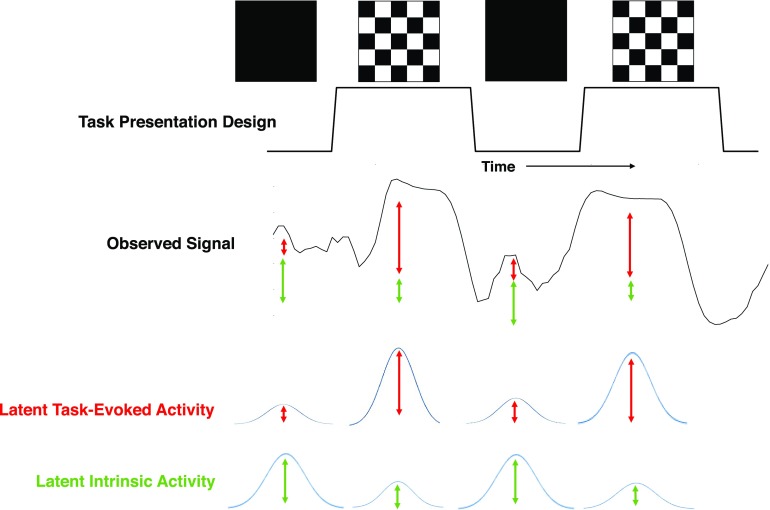
Conceptual illustration of the linear superposition principle. In this example, a participant is presented two sequences of fixation (rest) and visual checkerboard blocks, summarized by the block structure below the stimuli. The observed signal from a task-responsive brain region exhibits a predictable increase in BOLD activity during task blocks, followed by a decrease in activity during fixation/rest blocks. The linear superposition principle states that this observed signal is linearly composed of unobserved or latent intrinsic and task-evoked activity (along with possible random measurement error), whose contribution to the magnitude of the BOLD signal is illustrated by green and red arrows, respectively. During the task block, where we expect the task-evoked signal to be dominant, task-evoked activity contributes most to the observed signal, and during the rest/fixation period, intrinsic activity contributes most to the observed signal. Importantly, the linear superposition principle claims that these two signals linearly sum together to form the observed signal.

The functional separation of neural activity into intrinsic and stimulus-driven components is enforced by the proposed ability to empirically separate intrinsic and task-evoked activity signals by the linear superposition principle. The need for the linear superposition principle in conventional task-based neuroimaging arises from the question of how to most accurately model task-evoked functional brain activity when intrinsic activity is presumably occurring simultaneously (Fox, Snyder, Vincent, & Raichle, 2007). In other words, how does one model a brain region’s response to an onset of task stimuli when nonrandom intrinsic activity is occurring at that region simultaneously? The solution or assumption to date has been that functional task-evoked and intrinsic activity linearly superpose. In other words, the total activity observed at any given brain region is a linear sum of intrinsic and task-evoked activity (Figure 1). The advantage of this assumption is that variability in across-trial task-evoked activity estimates is accounted for by variability in the underlying intrinsic activity (Fox et al., 2007; Fox et al., 2006). Despite the variability in task-evoked activity due to intrinsic activity from trial to trial, averaging across trials should recover the “true” task-evoked response to the stimulus. Thus, because of the linear combination of these two sources of activity, and across-trial averaging of neural activity following the stimulus presentation, task-evoked activity can be isolated from intrinsic activity.

Previous studies suggest that the linear superposition of intrinsic and task-evoked activity may be true of functional neuroimaging signals (Arieli, Sterkin, Grinvald, & Aertsen, 1996; Becker, Reinacher, Freyer, Villringer, & Ritter, 2011; Fox et al., 2006; Schölvinck, Friston, & Rees, 2012). For example, by recording activity in the cat visual cortex using electrophysiological methods, Arieli et al. (1996) demonstrated that evoked responses from a single trial of visual stimuli could be predicted by a simple sum of the deterministic (averaged) response and the preceding ongoing activity. More recently, Schölvinck et al. (2012) demonstrated through a psychophysiological interaction (PPI) analysis of BOLD signals from the primary visual cortex and functionally connected voxels identified at rest, that the estimated intrinsic and task-evoked activity combined in a largely linear fashion. Thus, the linear superposition principle supposedly provides an easily understandable and empirically supportable account of how these two sources of the signal combine. However, we argue below that recent evidence challenges this conclusion.

The presumed separability of these signals has led to dichotomous interpretations of the functional brain processes that these signals represent. For example, a popular theoretical account of the functional significance of intrinsic activity recorded during nonstimulus blocks and resting-state scans is as a “default mode” of brain function (Gusnard & Raichle, 2001; Raichle et al., 2001). This default mode of activity (not identical to the default mode network) represents a state of the brain that is produced by the internal dynamics of the brain, which has little or no relationship to external stimulation. This default mode of brain function has been variously inferred to represent spontaneous cognition (Christoff, Ream, & Gabrieli, 2004; Fransson, 2006), memory consolidation (Duyn, 2011), or predictive coding for future information processing (Raichle, 2010). In contrast, task-evoked activity corresponds to changes in neural activity driven or caused by external stimulation, and represents direct stimulus processing.

## EVIDENCE CHALLENGING THE DICHOTOMY OF INTRINSIC AND TASK-EVOKED ACTIVITY

The distinction between intrinsic and task-evoked activity in terms of internally driven and stimulus-driven processing and the linear superposition principle provides an overall picture of these two types of activity as relatively independent and encapsulated components of the observed neural signal. In the following section, we detail why we believe that the division of neural activity into intrinsic and task-evoked activity is artificial, and not an actual division respected by neural processes. First, we present evidence demonstrating a close functional interdependence between intrinsic and task-evoked activity, and a much closer attunement of intrinsic activity to environmental stimuli and context. Second, we present research on neural signal variability that provides evidence that the linear superposition principle is not true of neural signals. Overall, we argue that this evidence challenges any sort of division of the neural signal into intrinsic and task-evoked components. Though we continue to refer to intrinsic and task-evoked activity throughout this section as if these were separate components of the neural signal, we go on to argue that these findings motivate an alternative understanding of brain activity with no reference to intrinsic and task-evoked activity as separate components of the neural signal.

### Empirical Studies of the Relationship Between Intrinsic Activity and Task-Evoked Activity

The conception of intrinsic and task-evoked activity as separate, independent components of the neural signal is contingent upon the view that these signals exhibit differing spatial and temporal properties. Differing spatial and temporal properties of these signals suggest that these forms of activity can be studied as separate functional processes, as envisioned by default-mode theories of intrinsic activity (Raichle, 2010). In this section, we challenge this view and describe empirical studies demonstrating that intrinsic and task-evoked activity exhibit similar temporal and spatial properties.

Research in animal models has found that spatiotemporal activity patterns observed during stimulus presentations are often observed in subsequent nonstimulus periods. In particular, several studies have found that neural activity patterns during stimulus presentation reverberate in neural activity following the stimulus. In a study using voltage-sensitive dye imaging to examine spontaneous activity in the mouse visual cortex, Han, Caporale, and Dan (2008) discovered that with repetitive presentation of visual stimulus, the evoked activity waves in response to a visual stimulus tended to recur in the intrinsic activity that followed the stimulus. Not only was this effect specific to the visual stimulus presented, it lasted several minutes without further visual presentation. Similar findings have been found in temporal patterns of single-unit recordings of the visual cortex in anesthetized cats (Yao, Shi, Han, Gao, & Dan, 2007). Yao et al. (2007) found that in examination of intrinsic activity following the presentation of time-varying natural scenes, an identical temporal spike pattern to that observed during the presentation of the scenes was observed several times. In a developmental study of visual activity using multiunit recordings in awake ferrets, Berkes, Orbán, Lengyel, and Fiser (2011) demonstrated that internal neural models of visual stimuli reflected in intrinsic activity progressively adapt to the statistics of natural visual stimuli over development. In other words, the internal predictive model represented by intrinsic activity had a greater match with the features of natural visual stimuli, represented by task-evoked activity in response to that stimuli, in older ferrets compared with young ferrets, suggesting that increasing age is associated with *a priori* internal models that are more adapted to natural visual stimuli.

In addition, intrinsic activity regimes in neuronal populations bear striking similarities to those task-evoked activity regimes driven by thalamocortical projections. Calcium imaging studies of large neuronal populations from mouse cortices (Cossart, Aronov, & Yuste, 2003; MacLean, Watson, Aaron, & Yuste, 2005) have found that stimulation of thalamocortical synapses, which are responsible for transmission of sensory signals, activates networks of neurons statistically indistinguishable from those observed during intrinsic activity, displaying both the same UP states (a depolarized membrane potential state of a population of neurons) and the same temporal sequence of activation patterns. Separate studies using high-resolution optical imaging of the visual cortex of anesthetized cats (Tsodyks, Kenet, Grinvald, & Arieli, 1999) have found that very similar patterns of population activity are observed when populations of neurons are “spontaneously” active, and when they are driven by visual stimuli. Similar patterns of activity are also observed in spatial covariance relationships measured by functional neuroimaging between intrinsic and task-evoked activity states. For example, studies examining functional connectivity differences between resting-state and task-driven states have found that despite small differences, functional connectivity patterns are strikingly similar between evoked and intrinsic states (Bertolero, Yeo, & D’Esposito, 2015; Bolt, Nomi, Rubinov, & Uddin, 2017; Cole, Bassett, Power, Braver, & Petersen, 2014; Finn et al., 2015; Smith et al., 2009). Taken together, these studies demonstrate that intrinsic activity, along with evoked activity, is dynamically attuned to the present environmental context, and that overall patterns of intrinsic activity are strikingly similar to patterns of task-evoked activity.

### Functional Significance of Intrinsic Activity

One potential justification for the separation of the neural signal into intrinsic and task-evoked activity is the idea that intrinsic activity corresponds to internal dynamics of the brain, and thus has no functional significance for behavioral output. However, most researchers now agree that the same task stimulus can have different neural and psychological effects depending on prestimulus intrinsic activity levels. This is particularly true in studies of the effect of oscillatory prestimulus activity on subsequent sensory experience in EEG and fMRI studies (Busch, Dubois, & VanRullen, 2009; Hesselmann, Kell, Eger, et al., 2008; Hesselmann, Kell, & Kleinschmidt, 2008; Lou, Li, Philiastides, & Sajda, 2014; Mathewson, Gratton, Fabiani, Beck, & Ro, 2009; Sadaghiani, Hesselmann, & Kleinschmidt, 2009; Sapir, d’Avossa, McAvoy, Shulman, & Corbetta, 2005; van den Berg, Appelbaum, Clark, Lorist, & Woldorff, 2016; van Dijk, Schoffelen, Oostenveld, & Jensen, 2008). In these studies, the prestimulus alpha-band level in the case of EEG or BOLD level in the case of fMRI is used to predict whether a randomly presented subliminal auditory or visual stimulus is consciously perceived. The common finding is that higher levels of baseline prestimulus activity are associated with a greater likelihood of consciously perceiving the stimulus during that trial. The assumption explicit in this research paradigm is that the intrinsic activity estimated by the prestimulus signal represents a sort of preparatory signal that prepares the brain for detection of a perceptual stimulus (Sadaghiani, Hesselmann, Friston, & Kleinschmidt, 2010). Thus, variation in the conscious perception of a subliminal stimulus is accounted for by variation in the level of the prestimulus signal, such that when the prestimulus level is high, the addition of task-evoked activity surpasses a threshold for conscious perception, and when the prestimulus level is low, the addition of the same task-evoked activity fails to reach the threshold for conscious perception. Similarly, Buckner (2010), in a case discussed in more detail below (see Conceptual Implications), demonstrates that the functional role of unit activity can depend on its relationship to ongoing background fluctuations.

Importantly, these studies highlight the functionally significant role of intrinsic activity in cognitive processing and behavioral output. Rather than simply reflecting static structural connectivity, historical coactivation patterns, or the internal dynamics of certain brain regions, intrinsic activity has a functionally significant effect on *within-trial* cognitive processing and behavioral output. These findings dismiss any notion of task-evoked activity as the sole signal of activity functionally relevant to task performance. On the contrary, at least in terms of behavioral output, the interplay between intrinsic and task-evoked sources of the observed signal is functionally relevant, and the full understanding of brain activity relevant to cognitive processing requires accounting for both of these sources.

### Summarizing the Findings So Far

What we see from neuronal recordings in animal models and functional neuroimaging of humans is that spatiotemporal properties of estimated intrinsic activity are very similar to those observed in estimated task-evoked activity (Berkes et al., 2011; Bermudez Contreras et al., 2013; de Lange, Rahnev, Donner, & Lau, 2013; Fiser, Chiu, & Weliky, 2004; Luczak, Barthó, & Harris, 2009). This extends to both the temporal dynamics and the spatial covariance relationships between populations of neurons or brain regions. These findings of similar spatiotemporal activity patterns suggest that similar functional processes are active during both periods of external stimulation and nonstimulation. In fact, below (see Moving Beyond the Intrinsic/Task-Evoked Dichotomy) we suggest that these findings are indicative of the spatiotemporal dynamics of a single functional process.

The functional significance of intrinsic activity is further confirmed by the behavioral significance of this activity for task performance. As described above, prestimulus intrinsic activity is predictive of subsequent perceptual processing. Thus, intrinsic activity, as measured by prestimulus BOLD signals, is functionally relevant for subsequent behavior. The sole focus on poststimulus task-evoked activity signals as important for understanding cognitive processing of an external stimulus disregards the functionally significant role of prestimulus intrinsic activity. In the section below on moving beyond the intrinsic/task-evoked dichotomy, we argue that researchers should attempt to describe this temporal trajectory of brain activity patterns from prestimulus to poststimulus without dividing this dynamic into intrinsic and task-evoked activity.

### Variability Reduction During Task-Driven States

While the empirical results highlighted above suggest that the separation of evoked and intrinsic activity by their differing spatiotemporal or functional properties is problematic, the greatest motivation for the separation of the neural signal into task-evoked and intrinsic activity with separate functional roles is the supposed independence of these two signals as assumed by the linear superposition principle. Previous studies (Arieli et al., 1996; Becker et al., 2011; Schölvinck et al., 2012) have provided support for the separation of these two components of the signal by demonstrating that these signals are additive, as assumed by the linear superposition principle. However, there is increasing evidence from studies of neural signals recorded by functional neuroimaging that the linear superposition principle is false, or at least not universal. We highlight findings from these studies below and discuss their implications.

As described above, the linear superposition principle maintains that the total activity observed at any given brain region is a linear sum of these two forms of brain activity. Work from He (2013) reporting that task-evoked and intrinsic activity *negatively* interact, however, calls the linear superposition principle into question. This work demonstrated that there is less task-evoked activity at higher baseline (or intrinsic) activity levels. By comparing trial-to-trial variability in BOLD activity between resting-fixation trials and visual-cue response trials and using an ingenious derivation of the Law of Variance, He (2013) found that a negative interaction between intrinsic and task-evoked brain activity was the only explanation (as opposed to a negative or positive interaction hypothesis) of the observed reduction in trial-to-trial BOLD variability between rest and task trials. In addition, this reduction in variability was behaviorally relevant: trial-to-trial variability in BOLD response among certain brain regions differed between fast and slow reaction time trials. For example, there was a large decrease in variability between slow and fast trials in the right cerebellum, which is consistent with its role in motor timing.

These results have been further corroborated by Huang et al. (2015), who demonstrate phase-dependent effects of spontaneous activity on task-evoked activity. Scheeringa, Mazaheri, Bojak, Norris, and Kleinschmidt (2011) similarly found that the magnitude of a visually evoked fMRI BOLD response in the early visual cortex depended on the current alpha phase amplitude in that area, a measure of intrinsic activity recorded using EEG. These findings were further anticipated by results demonstrating interactions of evoked and ongoing activity in neuronal burst firing and up-and-down states (Kisley & Gerstein, 1999; Petersen, Hahn, Mehta, Grinvald, & Sakmann, 2003). Variability reduction following the onset of task stimuli had been anticipated in earlier studies of neural signaling as well. Churchland et al. (2010) observed consistent declines in firing rate variability in spiking neurons following stimulus onsets, even with little changes in mean firing rate. Ponce-Alvarez et al. (Ponce-Alvarez, Thiele, Albright, Stoner, & Deco, 2013) have further shown that reductions in firing rate variability of direction-selective middle temporal neurons vary systematically with stimulus direction. Taken together, these findings suggest that intrinsic and task-evoked activity cannot be understood as isolated, additive components. Rather, the level of either intrinsic or task-evoked activity is crucially dependent on the other, and vice versa.

The concept of a nonadditive interaction between intrinsic and task-evoked activity can be illustrated in terms of a linear regression model with a multiplicative interaction term:

$$Y_{t}=\beta_{1}I_{t}+\beta_{2}T_{t}+\beta_{3}(I_{\text{t}}\times T_{t})+e_{t},$$

where *Y* is the neural signal at time *t*, *I* is the unobserved intrinsic activity at time *t*, *T* is the unobserved task-evoked activity at time *t*, (*I* × *T*) is the multiplicative interaction term modeling the dependency between the two forms of activity, *e* is the unexplained variance in the neural signal at time *t*, and *β*’s are the linear weights (assuming a linear relationship for the sake of argument) relating the three terms to the BOLD signal. According to the linear-superposition principle, there is no interaction between the two forms of activity (*β*_3_ = 0); each form of activity can be understood as a separable, additive component, in which the effect of one form of activity (*β*_1_ or *β*_2_) can be understood without the other, and vice versa. In the case of a nonzero interaction between the two forms of activity (*β*_3_ ≠ 0), the effect of one form of activity depends on the level of the other form of activity. In other words, *the relationship between task-evoked activity and the neural signal differs at different levels of intrinsic activity*. A nonzero interaction implies that the effect of either form of activity on the neural signal cannot be understood in isolation, but crucially depends on knowledge of the other.

## MOVING BEYOND THE INTRINSIC/TASK-EVOKED DICHOTOMY

### Conceptual Implications

The research studies summarized above demonstrate that intrinsic and task-evoked activity exhibit similar spatiotemporal properties, and that these two activity signals are not strictly separable. These empirical findings should motivate a conceptual reform. Importantly, intrinsic and task-evoked activity are *unobserved* or *latent* variables posited as the two systematic sources of the neural signal at any time point. Several researchers have attempted to empirically extract these two unobservable sources of the neural signal into separable, additive components (Arieli et al., 1996; Becker et al., 2011; Fox et al., 2007), and thus, separable brain functions. However, the validity of such an approach is contingent upon the independence of these two forms of activity from each other. The functional interdependence between these two forms of activity, as implied by stimulus-onset variability reductions in the neural signal, and their similar spatiotemporal properties, questions the utility of their distinction. Rather than interpreting this interaction phenomenon in terms of a conventional distinction between intrinsic and task-evoked activity, He (2013) suggests that there is only *one* sort of functional activity, the brain’s trajectory in a multidimensional functional space before and after stimulus presentation. In other words, if the effect on overall brain activity of a stimulus or task parameter depends on the background activity, and the effect of the background activity depends on the details of the stimulus or task, then trying to identify the functional meaning of evoked (or intrinsic) activity in isolation (i.e., judging the poststimulus increase in amygdala activation to be caused by the emotional content of the stimulus) is not just practically impossible, but scientifically dubious. Rather than try to identify the separate functional contributions of evoked and intrinsic activity, it would be better to treat brain activity as a single, unified, multidimensional variable in need of interpretation. A simple example from one of the most studied structures in the brain may illustrate the promise of a more integrated approach.

Some cells in the hippocampus display place selectivity under certain circumstances (O’Keefe & Nadel, 1978). In light of this observation, it is common in the literature to interpret activity in an individual hippocampal place cell as indicating the animal’s current location: firing in cell X means the animal is at location X. But in fact the matter is more complicated than this. As Buckner and colleagues have demonstrated (Buckner, 2010), these cells fire not just when an animal is at a given location, but just before, and just after, too. Interestingly, the differences between concurrent firing (the “you are here” signal), prospective firing (signaling in advance of being at a location), and retrospective firing (signaling after the animal has left a location) is marked not by any difference in the neuron’s activity itself, but rather by its relationship to the background theta-band (∼6–10 Hz) oscillation of the whole hippocampus. In its retrospective role, the cell fires earlier, and in its prospective role later, in the theta cycle than it does when the animal is actually at the location in question. In other words, what that cell’s activity *means*—the function it is serving, or the information it represents—depends on how that activity relates to the ongoing background oscillations.

As this simple example illustrates, thinking about brain activity in a more unified way means giving up on the idea that individual parts of the brain are always best understood as components with fixed functions. But is there a viable alternative? He’s observation that one effect of stimulus onset is a *reduction* in the variability of ongoing activity points to one promising option. It is a well-established finding that a reduction in the overall variability in the parameters defining the evolving state of a complex system is a sign of the dynamic construction of task-relevant synergies (Bernshteın, 1967; Latash, 2008). A synergy is a functional grouping of individual elements (e.g., neurons, muscles, limbs, or individuals) that are temporarily constrained to act as a single coherent unit (Kelso, 2009). In a synergy, the interacting elements mutually compensate for variation in the parts to reduce variability in the activity of the whole. For instance, Bernshteın (1967) observed that there is more variability at the joints of a blacksmith’s arm than there is in the location and trajectory of each successive hammer strike, because the blacksmith’s muscles had been temporarily unified into a variability-reducing synergy.

**Figure 2.**
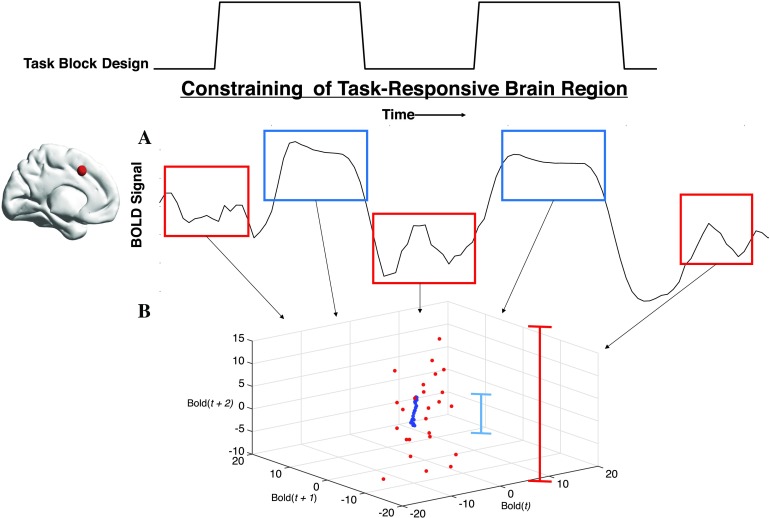
Temporal activity space and task response. Illustration of the temporal activity space approach used by previous research (He, 2013) to study a task-responsive brain region’s (e.g., dorsal anterior cingulate cortex; dACC) temporal trajectory across a task scan. (A) The time series of a hypothetical brain region (e.g., dACC) exhibits a consistent increase in signal amplitude to the task blocks. (B) The constraining of dACC’s temporal trajectory across the task scan is illustrated by the three-dimensional scatterplot. Using the same approach as He (2013), we plotted the BOLD amplitude at three randomly chosen successive time points—Bold(*t*), Bold(*t* + 1), and Bold(*t* + 2)—during the off-task (red) and on-task (blue) blocks. As can be observed, the dACC’s trajectory tightens during the on-blocks (i.e., smaller volume of space) and expands during the off-blocks (i.e., larger volume of space), as indicated by the length of the lines or whiskers next to the scatterplot.

In the current case, we should understand the observation of reduced variability in functional brain activity as evidence that the relevant parts of the brain are enacting a system of mutual constraints to move the whole into and to maintain a task-appropriate functional configuration. He (2013) suggests capturing these dynamic changes by projecting brain activity into a multidimensional functional space (Buonomano & Maass, 2009), and taking the brain’s trajectory through that space to represent its entry into different task-specific configurations as circumstances dictate. He (2013) illustrates this by projecting her measurements into a three-dimensional temporal activity space, as illustrated in Figure 2, and demonstrates that after the onset of task demands, the neural trajectory occupies a smaller three-dimensional volume than before the task-relevant constraints were enacted. While this analysis provides a powerful proof of concept, more data-driven, whole-brain approaches capable of giving a spatiotemporal description of these synergies across the course of a task scan (such as state-space models, Mastrovito, 2013, or multivariate temporal clustering algorithms, Omranian, Mueller-Roeber, & Nikoloski, 2015; Zhou, De la Torre, & Hodgins, 2008) may lead to additional insights.

The intrinsic/task-evoked dichotomy encourages functional neuroimaging researchers to separate this dynamical process into intrinsic and task-evoked components. Not only does this experimenter decision artificially separate this dynamical process, it provides researchers with the difficult task of putting these two components back together again. Instead, the methodological goal for experimenters should be to attempt to provide a holistic description of this dynamic process as it unfolds over time throughout the task, without resorting to the intrinsic/task-evoked dichotomy.

### The Brain and Predictive Processing

The separation of brain function into intrinsic and task-evoked components encourages a reflexive view of brain function (Raichle, 2010) in which the brain awaits stimulation from the environment. Thus, task-based functional neuroimaging researchers have often focused on the poststimulus neural signal as the primary carrier of information regarding cognitive processing. However, the evidence cited above suggests that the division of brain activity into intrinsic and task-evoked activity controlled by the onset of external stimuli is problematic. The alternative picture we present is a nondecomposable view of brain function where the brain is in constant adjustment and readjustment to maintain equilibrium with the environment (Kiverstein & Miller, 2015) in terms of the construction and maintenance of task-relevant synergies. In other words, the brain is not primarily reflexive or passive, but actively engaged with its environment, or *enactive* (Anderson & Chemero, 2016; Chemero, 2009; Noë, 2004), such that any state of the brain is one of action that is continuous, rather than separable into periods of internal mentation and stimulus-processing controlled by the onset of stimuli from the experimenter.

**Figure 3.**
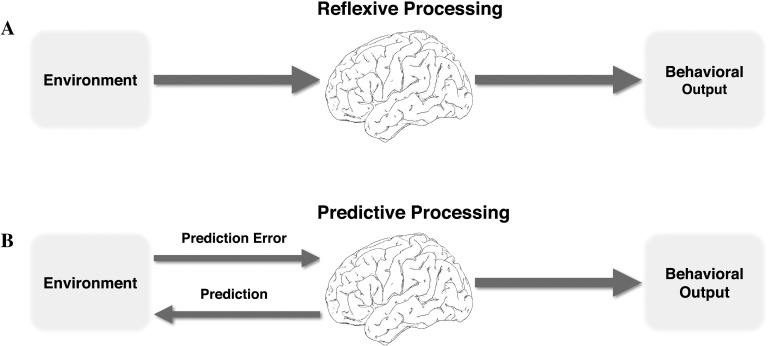
The reflexive and predictive accounts of brain function. (A) According to the reflexive, stimulus-driven processing account, environmental inputs (represented by the arrow pointing to the brain) are received by the brain for neural processing, with the subsequent production of a behavioral response (represented by the arrow leaving the brain). Thus, the brain’s interaction with the environment is governed by the onset of stimuli. (B) According to the predictive-processing account, the brain is neither a passive recipient of external stimuli nor exclusively dedicated to internal processing, but is constantly active, continually trying to predict the stream of sensory stimulation it receives from the environment (represented by the “prediction error” and “prediction” arrows moving to and from the brain and environment). Of note, behavioral output in many predictive-processing theories is intimately related to the sensory input (and prediction errors) received by the system and would be represented in the following diagram by feedback relationships from behavioral output to the brain and environment.

Predictive-processing theories of brain function offer a comprehensive interpretative framework with which to understand this dynamical process of adjustment and readjustment to the environment. Roughly, predictive-processing theories argue that the brain (or more accurately, the person as a whole) primarily interacts with the world through *prediction* or *expectation*. The brain’s predominant function in these theories is prediction-generation of incoming stimuli, such that the brain attempts to minimize the error between *prediction* or *expectation* of the incoming stimulus and the actual stimulus itself. In other words, the brain is not a passive receiver and processor of external stimuli, but constantly active, continually trying to predict the stream of sensory stimulation it receives from the environment (Clark, 2015), which is reflected in neural activity recorded both during prestimulus periods (and the so-called resting state) and during poststimulus periods. According to these models, neural activity recording during nonstimulus periods represents a sort of background activity preparing the individual for further processing of external stimuli (Clark, 2015). This preparatory neural activity is reflected in both the temporal dynamics of the signals across time, as well as the spatial patterns of activity averaged over time. In fact, static functional connectivity measurements averaged over time that show surprising stability across mental states and across time (Cole et al., 2014; Finn et al., 2015) might reflect a mindset, or a rough, coarse set of predictions (representing needs, goals, context-sensitive conventions, etc.) that are always brought to bear on incoming stimuli (Bar, 2011). The differences between the reflexive view of brain function and predictive view of brain function are illustrated in Figure 3.

The alternative account of brain function in terms of construction and maintenance of task-relevant synergies, and the description of this process in terms of the temporal trajectory of brain activity across time, suggests that the predictive-processing capacity of the brain is better understood in terms of dynamic engagement with the environment across time. Splitting this dynamic engagement into an intrinsic activity component and a stimulus-driven activity component obscures this process. As the empirical studies cited above demonstrate, the spatial and temporal properties of these hypothesized forms of activity are similar, suggesting that they reflect similar neural functions. We suggest that intrinsic and task-evoked activity exhibit similar properties because they are simply artificial divisions of the same functional process, the brain’s predictive engagement with the environment. A predictive-processing account describes the brain’s functional trajectory in terms of its spatial and temporal activity patterns across time as the person engages in a task, and attempts to provide a mechanical account of the systems-level, cellular, and molecular mechanisms that causally contribute to this temporal process.

The predictive-processing framework of brain function resonates with several systems-level theoretical approaches gaining popularity in neuroscientific research, including dynamical systems theory and the related coordination dynamics framework. A dynamical systems theory of the brain envisions the brain-body-environment as a complex system of interacting components moving in and out of steady or fixed states (Favela, 2014; Kiverstein & Miller, 2015). These systems are sometimes described in terms of their “critical” behavior (Beggs & Timme, 2012). Some systems with interacting components exhibit a property known as criticality, which is roughly a state of interaction in the system that emerges between total asynchrony (disorder) and total synchrony (order) of its components called a critical point. This sort of state exhibits nonglobal synchrony, but with local areas/modules of synchronous interaction among the components of the system. Importantly, models of critical systems have demonstrated beneficial properties of the critical state: efficient communication among system components (Beggs & Plenz, 2003), as well as an increased ability of the system to respond to inputs of different sizes, called dynamic range (Shew, Yang, Petermann, Roy, & Plenz, 2009). Studies have demonstrated that the brain at rest exhibits properties consist with a critical system (Haimovici, Tagliazucchi, Balenzuela, & Chialvo, 2013; Meisel, Olbrich, Shriki, & Achermann, 2013). However, consistent with the results of He (2013), Fagerholm and colleagues (2015) found that the brain exhibits *subcritical* dynamics during a focused-attention task. A subcritical dynamic corresponds to more global synchrony and a *lower* dynamic range (i.e., a reduced ability to respond to different system inputs). These results are consistent with an increased need for tuning out task-irrelevant information during cognitive task performance. Understood from a predictive-processing perspective, the movement of the neural system in and out of steady or fixed states does not occur simply as the result of external stimulation. In fact, as shown above, the organizational state of the neural system facilitates or inhibits the processing of incoming external stimulation. Thus, successful cognitive performance relies on an adequate dynamic configuration of the system before the onset of relevant external stimuli, as opposed to simple adjustments after the fact.

Our theoretical framework is also consistent with the theoretical framework of coordination dynamics and the related notion of metastability (Tognoli & Kelso, 2014), both intimately related to dynamical systems theory. In the coordination dynamics framework, the brain is modeled as a complex system of neural processes at multiple temporal and spatial scales that exhibits both self-organizing tendencies and imposed organization due to external constraints on the system. According to some coordination dynamics theorists (Kelso, 1995, 2009; Kelso & Tognoli, 2009; Tognoli & Kelso, 2014), functional brain processes exist at rest in a metastable state, or a state of nonglobal synchrony involving simultaneous integration and segregation over space and time. Metastable coordination dynamics leads to a system that is not reflexive or stimulus-driven, but capable of flexible function in the absence of input. In fact, spontaneous metastability may provide the basis for observations of dynamic functional connectivity, or time-dependent changes in functional connectivity estimates, at rest (Deco, Tononi, Boly, & Kringelbach, 2015; Hutchison et al., 2013). In the case of a cognitive task, task demands and external inputs to the system would result in a phase transition of the system to a more globally ordered, synchronous state that would be necessary for adequate task performance. In line with a predictive-processing framework, the functional state of the system is not simply imposed by the external environment, but is self-organized. The relevant data for functional neuroimaging research should not be the spatiotemporal functional organization after task onsets, but how task onsets change the spatiotemporal properties of an already established functional organization.

### Mechanistic Description of This View of Brain Activity

As demonstrated by several researchers (Cossart et al., 2003; MacLean et al., 2005; Tsodyks et al., 1999; Yao et al., 2007), the cortical activity that results from thalamic sensory projections may be dependent upon already predefined neural circuits. The fact that projections from thalamic fibers give rise to responses matching spontaneous activity suggests a mechanistic interpretation of our view in terms of attractor models in neural networks (Petersen, 2005). Attractor states represent patterns of neural activity to which the brain tends to maintain and return to across time. Envisioning brain activity in terms of dynamic shifting between attractor states across time explains the similar temporal and spatial activity profiles between so-called intrinsic and task-evoked activity. Sensory stimuli from the environment can move the brain into and out of different attractor states, but these attractor states reoccur during periods of no external stimulation as well. According to our view of brain activity, the transition between attractor states is a mechanical implementation of the brain’s predictive capacity to anticipate incoming sensory stimuli and enact or fine-tune a given task-relevant synergy. For example, over the course of a task involving repeated stimulus presentations of the same visual category type (e.g., animals), the brain may begin to exhibit a tendency to move toward a conducive attractor state that prepares the individual for future engagement with the presentations of the same category. This is consistent with observations of temporal and spatial reverberation of activity patterns observed in a preceding visual stimulus presentation (Han et al., 2008; Yao et al., 2007).

While these findings are derived from cellular recordings in animal models, a potential connection to functional neuroimaging is the fact that the neural activity of these attractor states is predominantly reflected in UP and DOWN fluctuations in neural membrane potentials (Cossart et al., 2003; MacLean et al., 2005; Petersen, 2005). UP and DOWN fluctuations of membrane potentials are members of a class of neural signals often referred to as slow-cortical potentials (He & Raichle, 2009; Raichle, 2006, 2010) that have been shown to be associated with the BOLD signal. Thus, we suggest that fMRI activity reflects the low-frequency transition between these attractor states. Separating the recorded activity, reflecting these temporal changes between attractor states, into intrinsic and task-evoked activity artificially divides this dynamic. What is of interest is the temporal trajectory of the brain’s attractor state transitions during the entirety of the task, rather than division of these dynamics into dichotomous pieces.

These states are most likely reflected in the emergence and stabilization of synchronous brain activity patterns, as well as whole-brain *temporal* patterns. Related to the temporal activity space concept above (Figure 2), the emergence and stabilization of these spatiotemporal regimes reflects the enaction of neural synergies that constrain (i.e., reduce the variability of) the brain’s activity space to certain spatial and temporal configurations. As described above, these neural synergies guide and prepare the brain for future action and facilitate processing of incoming stimuli. The goal of functional neuroimaging research in this account is to detail both the spatial and the temporal whole-brain activity patterns that facilitate successful cognitive performance, and how these existing patterns are changed by the onset of experimental stimuli. While these synergies cannot be represented by the simple conceptual analysis in Figure 2, we discuss methods below that are capable of capturing these spatiotemporal dynamics.

## A NEGLECTED FORM OF FUNCTIONAL RELATIONSHIP IN THE BRAIN: ENABLING CONSTRAINT

Many may see this sort of description of brain function as foreign to the traditional cognitive neuroscience explanation involving the ordered flow of information between cognitive components (e.g., visual form recognition to visual object identification) corresponding to different brain regions or brain networks. Cognitive neuroscience often pursues mechanistic explanations of cognitive processes in terms of the decomposition of those processes into subfunctions, and the mapping of those subfunctions onto different brain regions or networks. The description of brain function as the construction and maintenance of task-relevant synergies, and its description in terms of a temporal trajectory of brain activity across time, is characteristically different from this sort of mechanistic explanation. This commitment to a decompositional theory of mechanistic explanation, what we call a componential mechanistic explanation (CME; Anderson, 2014; Craver & Bechtel, 2007), may explain why some researchers have rejected dynamical system accounts of human cognition as explanatorily irrelevant (Botvinick, 2012; Eliasmith, 2012; Howes, 2012; Wagenmakers, van der Maas, & Farrell, 2012), and why cognitive neuroscience has chiefly played the role of discovering where in the brain these processes occur, and deciding between competing theories of how they occur. In this section, we discuss CME-style explanations in cognitive neuroscience, and introduce a type of mechanistic explanation, known as enabling constraint, that better captures our theory of brain function.

### Mechanistic Constitution Relationships in Cognitive Neuroscience

CME-style explanations are best captured in terms of so-called box and arrow models that are characteristically illustrated using boxes to represent each area or network of the brain that performs some prescribed process and arrows to indicate the flow of information between them. This tradition of CME explanation is strong in cognitive neuroscience and was inherited from cognitive psychology’s long tradition of CME, most succinctly formalized by Sternberg (1969). According to Sternberg, cognitive psychology and more recently, cognitive neuroscience, is in the business of detailing the chain of processes that transform a stimulus into a response. These processes can be further decomposed into mechanistic stimulus-processing-response chains and so forth. Thus, a good explanation of a cognitive process involves decomposing the system responsible into parts and showing how those parts organize together to produce the cognitive process (Craver & Bechtel, 2007). This inheritance from cognitive psychology explains why cognitive neuroscience has chiefly played the role of detailing where in the brain these processes occur, and deciding between competing theories of how they occur. But despite the enormous productivity of this explanatory approach, it doesn’t capture all possible explanatory relationships of the brain and may reinforce popular dichotomies (e.g., task-evoked/intrinsic activity).

### Enabling Constraint

The alternative picture of brain function developed here does not easily lend itself to standard componential explanations of function. However, it *is* naturally captured by another style of mechanistic explanation called *enabling constraint* (Anderson, 2014). The notion of enabling constraints is relatively new to the cognitive neurosciences, but it may prove to be an important conceptual tool. An enabling constraint is a relationship between entities and/or mechanisms at a particular level of description and a functional system at the same or a different level, such that the entities/mechanisms bias (i.e., change the relative probabilities of) the outcomes of activity in the system. Each element of a complex system has multiple causal capacities, and the mutual constraints imposed by their organization serves to simultaneously limit those capacities and enable specific function (Anderson, 2014). For example, relations between a system’s parts (at some level of description) may serve to suppress a subset of that system’s behavior, thus inducing selectivity.

Here, we see a clear connection to the above descriptions of brain activity in terms of the construction of task-relevant synergies. At any state (evoked or intrinsic), the brain’s activity space is *constrained* so as to enable certain functions and suppress others. Thus, during a focused-task state (e.g., Fagerholm et al., 2015) there is an increased need for the system to organize itself in such a way as to filter out task-irrelevant features of the environment. However, there is no implicit idea of ordered flows of information between neural regions at different processing stages. Rather, the enabling constraints on the system offer the necessary conditions under which these flows of information can occur, and bias this flow to achieve a predictive equilibrium with the environment. It is worth emphasizing that there is no inherent contradiction between CME and enabling constraints in cognitive neuroscience; instead these two types of explanation complement each other, and both will likely prove to be necessary in the study of brain function. For example, the ordered flow of visual-sensory information within the visual system (primary visual cortex to secondary visual cortices) would naturally be accounted for in terms of a componential explanation between regions of the cortex. However, this information flow takes place within a larger system of functional constraints (Fagerholm et al., 2015; He, 2013; Scheeringa et al., 2011) that bias or constrain this flow of information, which would naturally be understood in terms of *enabling constraint* relationships. Describing these enabling constraint relationships and how they relate to the predictive-processing functions of the brain requires systems-level neuroscientific methods that can adequately capture the dynamics of these relationships as they unfold over time.

The relevant relationships identified in *enabling constraint* explanations can be broadly divided into functional and structural constraints on a neural system. Structural constraints, such as structural networks of fiber tracts between areas of the cortex, would represent *strong* constraints on a neural system, limiting neural communication to an anatomical backbone of communication pathways. Functional constraints represent transient*weak* constraints on the neural system attuned to environmental and stimulus contingencies, which are themselves dependent on structural/anatomical constraints of the neural system (Honey et al., 2009; Honey, Thivierge, & Sporns, 2010; Mišić et al., 2016; Park & Friston, 2013; Toosy et al., 2004). For example, transient periods of spatiotemporal activity patterns that constrain lower-level processing in individual brain regions represent a type of functional constraint. Importantly, structural/anatomical constraints on the system are not invariant over time, and themselves show long-term plasticity to environmental changes (Burke & Barnes, 2006; Jones et al., 2006; Ramachandran & Rogers-Ramachandran, 2000; Reiter & Stryker, 1988; Woolf & Salter, 2000). Thus, both structural and functional constraints are dynamic over time, at larger and smaller temporal/spatial scales, respectively. Understanding the cognitive capabilities of the brain will mean detailing the variety of nested long-term structural and short-term functional constraints relevant to the production of that capability. Functional neuroimaging plays a role in this area by detailing the myriad of spatial and temporal patterns of neural activity, and associating these patterns with unique task-relevant synergies critical for different task-relevant processing.

### Future Directions

#### Changing current methodological practices

The alternative perspective of functional brain activity presented here encourages new thinking in functional neuroimaging and opens new avenues for productive lines of research. However, the current division of functional neuroimaging research, particularly fMRI research, into resting-state and task-based research paradigms is an obstacle to progress. Researchers using resting-state approaches have typically focused on the application of multivariate techniques to study spatial covariance patterns among brain regions during periods of no external stimulation, and linked these to cognitive, disease, and behavioral variables. Researchers using task-based approaches have traditionally focused on the task-evoked activity following stimulus presentation (typically using some form of the general linear model; Friston et al., 1994), and linked these to theories of cognitive processing, as well as disease and behavioral variables. In practice, it is possible that a major motivation for separating the neural signal into task-evoked and intrinsic components is due to this division in functional neuroimaging research, and the limited availability of methodological approaches for studying both of these states simultaneously. Both research paradigms initially assumed a static view, in which the brain processes reflected by these activity patterns can be represented in a single pattern of task-evoked response across the brain, or a single pattern of functional connectivity relationships averaged across time. Dynamic multivariate techniques are needed to describe and explain brain process in terms of the evolution of spatiotemporal activity patterns across time. Most importantly, activity recorded during evoked and intrinsic periods as studied by task-based and resting-state researchers, respectively, are both crucial data points in the study of cognition and are more informative when studied *together*, rather than separately.

Importantly, we do not suggest that functional neuroimaging studies of the differences in the spatiotemporal organization of the brain during task and resting states are insignificant (Bolt et al., 2017; Calhoun, Kiehl, & Pearlson, 2008; Cole et al., 2014; Smith et al., 2009), nor do we suggest that research within the realm of task and resting-state research is uninformative. These studies provide a wealth of information regarding the spatiotemporal activity patterns of both states. We recognize that there is utility in isolating spatiotemporal activity patterns that are the result of stimulus presentations. We likewise see the value in characterizing recurring synchronous patterns of intrinsic activity. However, we believe that a more comprehensive understanding of brain function can result from acknowledging the functional and dynamic interdependence of these signals, and studying spatiotemporal patterns of activity across these states. Studies of the relationships between these different experimental states can give us an understanding of how external stimulation “moves” the brain in and out of task-relevant synergies. However, we do caution that such explanations of these differences should be understood from a holistic perspective, emphasizing the functional interrelationships among the spatiotemporal activity patterns recorded during each state.

Fortunately, the development of techniques for studying the characteristics of activity patterns across time are growing increasingly popular and are bridging the divide between resting-state and task-based researchers. For example, dynamic functional connectivity techniques (Allen et al., 2014; Chang & Glover, 2010; Hutchison et al., 2013) measure dynamic changes in functional connectivity within full-length resting-state fMRI scans. Measurements of dynamic functional connectivity relationships during task states have found that these connectivity patterns dynamically adjust to external stimuli and task demands (Cole et al., 2013; Gonzalez-Castillo et al., 2015; Krienen, Yeo, & Buckner, 2014; Kucyi & Davis, 2014; Shine et al., 2016). For example, using a time-resolved network analysis, Shine et al. (2016) demonstrated that the brain’s functional organization dynamically shifts from a segregated state of tightly interconnected functional modules to an integrative state with functional communication across functional modules, and that these dynamics are related to ongoing cognition.

While there is no single functional neuroimaging analysis that is best suited for describing the dynamical process of spatial and temporal patterns of neural activity across time, the broad class of systems-level neuroimaging analysis techniques, including dynamic network analysis (Allen et al., 2014; Bassett et al., 2011; Shine et al., 2016), dynamical systems analysis (Fagerholm et al., 2015; Kelso, 1995; Tognoli & Kelso, 2014), state-space modeling (Eavani, Satterthwaite, Gur, Gur, & Davatzikos, 2013; Janoos, Singh, Machiraju, Wells, & Mórocz, 2011; Suk, Wee, Lee, & Shen, 2016), and temporal clustering algorithms (Omranian et al., 2015; Zhou et al., 2008) provide the most potential for describing and explaining the functional organization and temporal dynamics of task-relevant synergies. We expect these techniques to continue to grow in popularity in the functional neuroimaging community and to provide further insights into the brain’s predictive engagement with its external environment.

These methods map clearly onto the enabling constraint explanatory account discussed above. Rather than segmenting the functional organization of the brain into separable components and determining how these come back together again, these methods describe the interactions and dynamics of the system considered as a whole. Within the enabling constraint framework, these methods support a mechanistic account of brain function without recourse to the focus currently dominant in cognitive neuroscience. Nevertheless, we believe that componential explanations and constraint-oriented accounts are complementary, as an adequate account of brain function requires attention both to local processing, and to the various systems-level constraints placed upon the parts of the system. Systems-level analysis methods will be crucial in this explanatory context.

#### Future empirical research directions

To provide an example of how this alternative conception of brain activity might provide insights into a particular content area of cognitive neuroscience, consider the study of the neural mechanisms of cognition using fMRI. The removal of the distinction between intrinsic and task-evoked activity, in terms of separable brain functions, means that prestimulus BOLD signals are a crucial data point in need of interpretation in any cognitive task paradigm. Rather than reflecting noncognitive intrinsic processing, these signals are crucially a part of the brain’s interaction with the external environment. For example, judging the poststimulus increase in BOLD activity in an attention task as corresponding to attentive processing fails to capture the functional relevance of the prestimulus temporal dynamics and functional organization. In fact, as demonstrated in the studies cited above (de Lange et al., 2013; Mathewson et al., 2009; Sadaghiani et al., 2010; Schölvinck et al., 2012), prestimulus BOLD magnitude is a crucial factor in conscious visual perception. Rather than a search for spatial patterns of significant poststimulus increases in BOLD activity, cognitive neuroscientists should open their investigations and analyses to the temporal and spatial patterns of BOLD activity *across time* during both prestimulus and nonstimulus epochs. The construction of an adequate task-relevant synergy for successful cognitive performance requires *both* a “correct” prestimulus temporal and spatial organization for prediction of the incoming stimuli, and a “correct” adjustment and attunement of this organization following stimulus presentation. Examination of both these periods of interest will be crucial for an adequate understanding of the neural mechanisms of cognition.

A potential area for future research in cognitive neuroscience is the transition point at which incoming sensory signals meet prestimulus or ongoing activity. In particular, how are incoming sensory signals incorporated into both the ongoing temporal dynamics and functional integration of the neural system? As demonstrated by He (2013), prestimulus and poststimulus activity can interact in surprising ways, not accounted for by a simple linear relationship between the two. Predictive-processing theories predict that repeated presentations of task stimuli should be incorporated in an identifiable way in the temporal and spatial neural dynamics of nonstimulus epochs. In fact, it is possible that the increased predictably of task stimuli is reflected in an increased match between temporal and spatial dynamics of stimulus and nonstimulus epochs, consistent with previous findings in multiunit recordings (Berkes et al., 2011). Ultimately, for this alternative research perspective to be further developed and refined, it will take bold and creative new research willing to challenge the long-held evoked/intrinsic assumption.

## FUNDING INFORMATION

This work was supported by the National Institute of Mental Health (R01MH107549) and a NARSAD Young Investigator Award to LQU.

## AUTHOR CONTRIBUTIONS

Taylor Bolt: Conceptualization; Writing – original draft. Michael L. Anderson: Conceptualization; Writing – review & editing. Lucina Q. Uddin: Conceptualization; Writing – review & editing.

## TECHNICAL TERMS

Intrinsic/evoked activity dichotomy: The view that the observed neural signal at any given time point is a linear combination of two unobserved sources, intrinsic and evoked activity.
Intrinsic activity: One of two latent sources presumed to be present during both stimulus and nonstimulus periods that represents the nonstimulus-related background activity.
Evoked activity: One of two latent sources of activity presumed to “add” on top of the ongoing intrinsic activity in response to stimulus presentation.
Linear superposition principle: The theory that intrinsic activity and evoked activity sum together linearly (i.e., simple addition) to produce the observed signal.
Predictive processing: The theory that the human brain’s predominant function is predicting the stream of sensory information it receives from the environment, such that the error is minimized between the prediction of the incoming stimuli and the actual stimulus itself.
Enabling constraint: A relationship between entities and/or mechanisms at a particular level of description and a functional system at the same or a different level, such that the entities/mechanism bias the outcomes of activity in the system.
Multiplicative interaction: A relationship between two variables (*A* and *B*), where the values of one variable (*A*) depend on the values of the other variable (*B*), and vice versa.
Stimulus-onset variability reduction: The finding that the onset of a stimulus produces a reduction in within-trial and across-trial variability of the signals in task-related neurons or brain regions.
Neural synergy: A functional grouping of neural regions that are temporarily constrained to move the neural system into and out of task-appropriate functional configurations.
